# Impact of Strain Engineering on Antiferroelectricity in NaNbO_3_ Thin Films

**DOI:** 10.1021/acsomega.3c01327

**Published:** 2023-06-20

**Authors:** Thorsten Schneider, Juliette Cardoletti, Philipp Komissinskiy, Lambert Alff

**Affiliations:** † Institute of Materials Science, Technische Universität Darmstadt, Alarich-Weiss-Strasse 2, 64287 Darmstadt, Germany

## Abstract

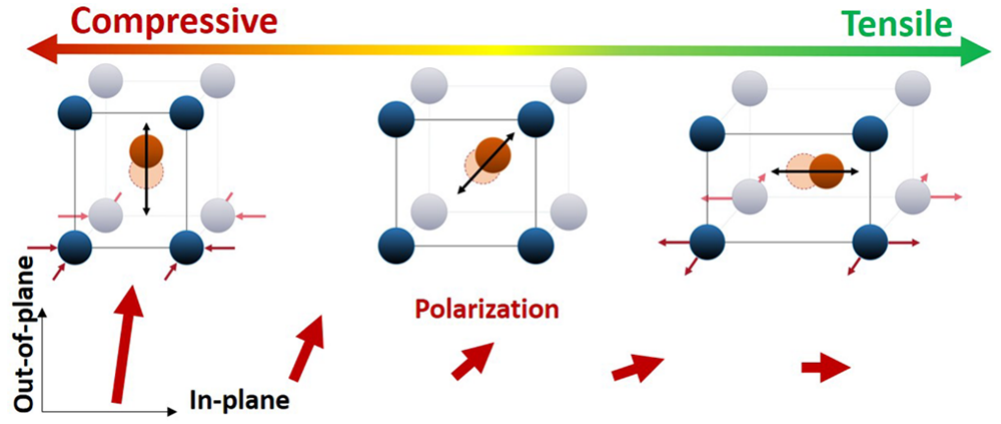

Thin films of NaNbO_3_ were grown on various substrates to investigate the effect of epitaxial strain on their structural and electrical properties. Reciprocal space maps confirmed the presence of epitaxial strain from +0.8% to −1.2%. A bulk-like antipolar ground state was detected via structural characterization for NaNbO_3_ thin films grown with strains ranging from a compressive strain of 0.8% to small tensile strains, up to −0.2%. For larger tensile strains on the other hand, no indication of antipolar displacements can be detected, even beyond the relaxation of the film at larger thicknesses. Electrical characterization revealed a ferroelectric hysteresis loop for thin films under a strain of +0.8% to −0.2%, while the films under larger tensile strain showed no out-of-plane polarization component. However, the films with a compressive strain of 0.8% present a saturation polarization of up to 55 μC·cm^–2^, more than twice as large for films grown under conditions with small strain, which is also larger than the highest values reported for bulk materials. Our results indicate the high potential for strain engineering in antiferroelectric materials, as the antipolar ground state could be retained with compressive strain. The observed enhancement of the saturation polarization by strain allows for substantial increase of energy density of the capacitors with antiferroelectric materials.

## 1 Introduction

Strain engineering has been established in many scientific fields to tailor and optimize desired properties as well as create new functionalities in devices.^1−5^ One major success is the improvement of ferroelectric (FE) properties, where percent levels of strain have the potential for a colossal increase in relevant quantities, such as saturation polarization and remanent polarization.^1,4^ Thus, strain engineering is a promising approach to extend to further research fields, especially ones closely linked to the field of FE, such as antiferroelectricity (AFE). Research into AFE has been intensified in recent years due to a number of potential applications, primarily in short-term energy storage and pulse power applications.^6−8^ However, only few publications directly address the influence of strain on AFE.^9,10^ Thus, despite the close relation between AFE and FE, a thorough understanding has not yet been achieved concerning the effect of strain on AFE performance.

Among the few known AFE materials, NaNbO_3_ has received much attention as a possible replacement of lead zirconate titanate solid solutions (PZT).^11,12^ Despite featuring the best AFE performance, it contains harmful lead, and thus, lead-free alternatives are desired.^6,13^ Furthermore, the transition between the AFE and FE phases in NaNbO_3_ is readily studied due to the small energy difference between the two phases.^14,15^ As a result, there is a coexistence of both phases in bulk materials.^16,17^ Thus, small changes to the energy landscape lead to a direct shift of the ground state. This shift can be identified by structural characterization, due to the associated change of the structural symmetry between the two phases: while the AFE phase (*Pbcm*) exhibits a unit cell with a quadrupled lattice parameter in one direction compared to the basic perovskite unit cell, due to a combination of ion displacements and octahedral tilts, the FE phase (*Pmc*2_1_) only reveals a cell doubling.^8^ Hence,  superlattice peaks unambiguously reveal the presence of the AFE phase.

The main strategy to affect the AFE-FE phase balance is via doping, where, so far, an empirical connection between the Goldschmidt tolerance factor of perovskites and the stabilization of the AFE phase has been established.^14,18−20^ Strain engineering is another option to manipulate the phase balance, as the AFE effect is accompanied by a strain response.^9^ On top of that, it also offers the advantage to tailor properties like the saturation polarization to increase the recoverable energy density.^4^ Furthermore, strain engineering can be combined with the doping approach and, hence, lead to a substantial increase in energy storage density. Methods to introduce strain into the material are arduous for bulk ceramics, especially if tensile strain is of interest.^9^ Hence, a thin film approach was chosen, as the type (compressive or tensile) as well as the level of epitaxial strain is promoted via choice of substrate. Moreover, thin films also have the advantage of higher electric breakdown strengths, which makes the investigation of electrical properties more accessible.^21,22^

The orthorhombic lattice parameters of NaNbO_3_ can be recalculated to the pseudocubic notation:^23^*a*_pc_ = 3.881 Å, *b*_pc_ = *c*_pc_ = 3.915 Å. These lattice parameters are calculated based on the AFE ground state,^24^ for which the quadrupling of the cell occurs in the direction of *a*_pc_. For the growth of NaNbO_3_, many isostructural perovskite substrates are available with lattice parameters suitable for inducing either compressive or tensile strain in NaNbO_3_ thin films, as shown in Table 1.

**Table 1:** Lattice Parameters of NaNbO_3_, LaNiO_3_,^25^ and the Substrates Investigated in This Work^a^

material	*a*	*b*	*c*	strain
	(Å)	(Å)	(Å)	(%)
NaNbO_3_	3.881	3.915	3.915	
LaNiO_3_	3.84	3.84	3.84	
LSAT	3.868	3.868	3.868	+0.77
SrTiO_3_	3.905	3.905	3.905	+0.26/–0.18
DyScO_3_	3.945	3.943	3.943	–0.72
GdScO_3_	3.965	3.961	3.961	–1.17

a For non-cubic perovskites, the corresponding pseudo-cubic lattice parameters were calculated.^26^ The last column indicates the induced strain for epitaxial growth of NaNbO_3_. For this calculation, it was assumed that the orientations align such that the lowest mismatch is achieved and the LaNiO_3_ layer is considered to be fully strained. Note that, in the case of SrTiO_3_, the strain calculation depends on the growth orientation, leading to either tensile or compressive strain.

Within the scope of this study, NaNbO_3_ thin films were grown with varying epitaxial strain, from compressive to tensile. Detailed structural investigations reveal a strain dependence of the ground state, with an antipolar ground state for compressive strains and the absence of antipolar displacements for growth on tensile strain-inducing substrates. The analysis of the polarization versus electric field behavior reveals no further stabilization of the AFE state, however, it shows a promising increase in saturation polarization for the case of compressive strain.

## 2 Experimental Section

The NaNbO_3_ thin films investigated in this work were produced via pulsed laser deposition (PLD). Details of the fabrication can be found in a previous report.^2^ After initial optimization of the film properties on SrTiO_3_(100), the same deposition parameters were utilized to grow NaNbO_3_ on the other substrates, (LaAlO_3_)_0.3_(Sr_2_TaAlO_6_)_0.7_(100) (LSAT), DyScO_3_(110) and GdScO_3_(110). These substrates were selected such that the evolution of the properties from compressive strain (LSAT) to tensile strain (DyScO_3_ and GdScO_3_) can be investigated (see Table 1). For each substrate, a series of films with increasing thickness from 10 to 400 nm was grown to study the impact of strain and critical thickness of relaxation. For the electrical characterization, a bottom electrode consisting of 25 nm thick LaNiO_3_ was grown before the NaNbO_3_ directly onto the substrate, also via PLD. While the out-of-plane lattice parameter of LaNiO_3_ appears to vary for different films, this layer has been shown to be fully in-plane strained and matching the orientation of the substrate.^2^ A parallel plate capacitor structure was fabricated via photolithography and sputtering of gold contacts, resulting in well-defined circular top electrodes with varying diameter from 0.002 to 0.78 nm^2^ and a thickness of 500 nm. The structural analysis was carried out using X-ray diffraction (XRD) (SmartLab, Rigaku) to characterize crystal quality, phase, and strain of the grown thin films. Leakage current characterization was carried out with a semiconductor characterization system (SCS) (4200-SCS, Keithley Instruments). The LaNiO_3_ bottom electrode was kept grounded and a voltage bias was applied to the Au top contact. The polarization response to an external electrical field was probed with a ferroelectric tester (TF2000, aixACCT Systems GmbH), optimized for thin films characterization. For this measurement, the field was applied from the bottom electrode and the top electrode was utilized for the current measurement.

## 3 Results and Discussion

**Figure 1:**
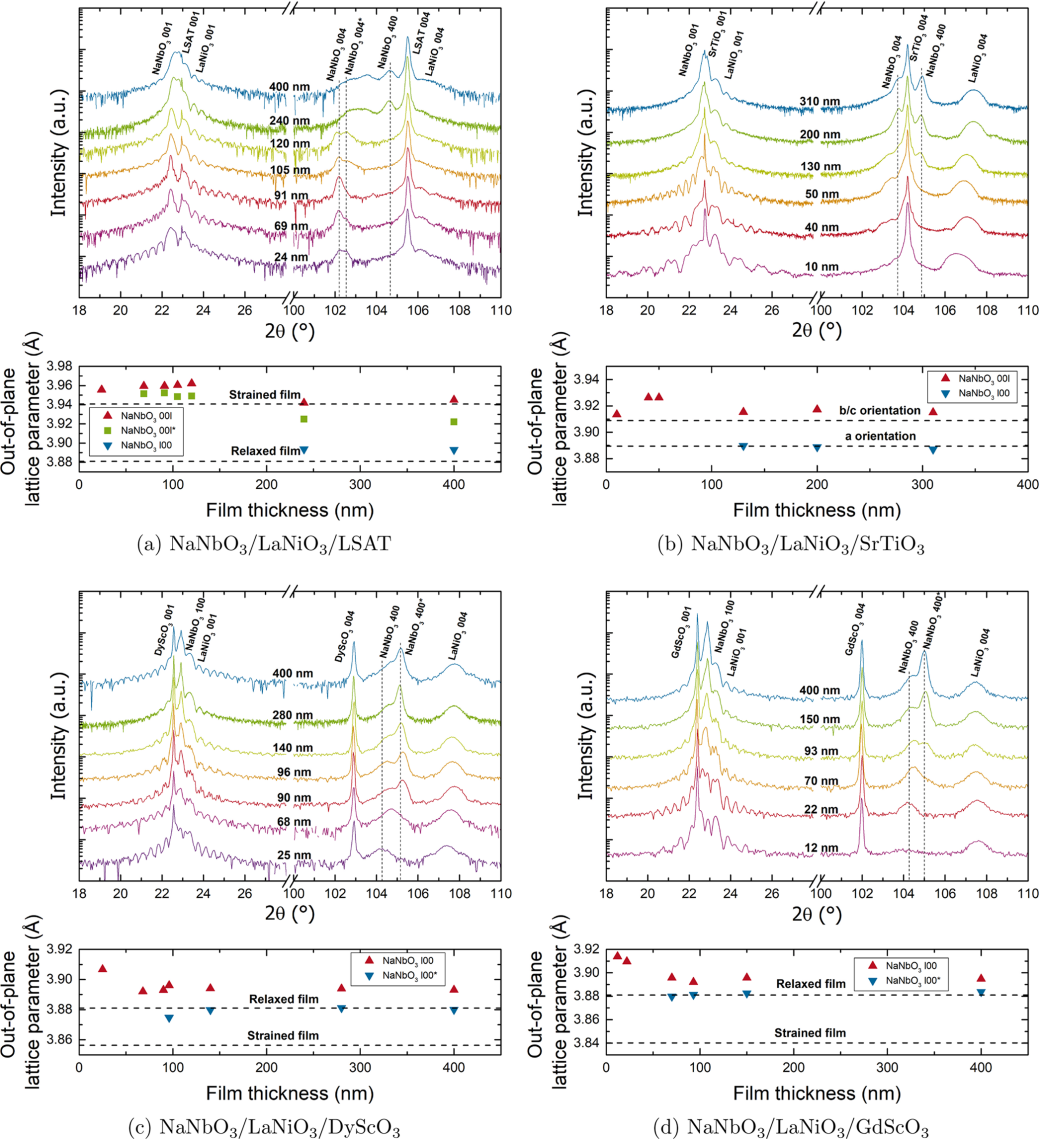
θ–2θ scans of NaNbO_3_ thin films for growth on different substrates. Shown are the regions around the 001 and 004 substrate reflections and the extracted lattice parameters from these scans. For the latter, the different contributions of NaNbO_3_ are marked with the corresponding orientation. For multiple reflections related to one orientation, the additional one is marked with a “*”.

In Figure 1, the 001 and 004 XRD reflections of the films and substrates are shown to visualize the evolution of the structure. The 001 reflection indicates the high quality of the films, as evidenced by the extensive Laue oscillations for the thinner films, while the 004 reflection is shown to better distinguish between different phases and orientations. The Laue oscillations were analyzed to extract the thicknesses of the films, which are indicated in the respective graphs. For thicker films, where the Laue oscillations could not be resolved anymore, the thickness was calculated based on the number of the laser pulses, using the growth rate calibration based on the thinner films.

Focusing on the 004 reflections, for each substrate, a transition can be found from the presence of one reflection for the NaNbO_3_ thin film to the appearance of other contributions. The corresponding NaNbO_3_ lattice parameters were extracted by fitting of the film 00l/l00 reflections and applying the Nelson–Riley method for correction. The resulting values are displayed below the XRD scan of the respective film–substrate combination. In these graphs, the lattice parameter of the bulk structure, as well as the expected lattice parameter for a fully strained film, based on the Poisson effect, are shown as dotted lines. The latter was calculated via the following formula derived from the general strain–stress relation:
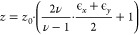
1where *z* is the final out-of-plane lattice parameter, *z*_0_ the bulk lattice parameter, ϵ_*i*_ are the strains in the corresponding direction *i*, and ν is the Poisson ratio. The latter was approximated as 0.3, based on similar perovskite materials.^27^ For the case of growth on SrTiO_3_ (Figure 1b), instead of the strained and unstrained values for the lattice parameter, the strained lattice parameters of the different orientations are given, as no relaxation occurs.^2^

**Figure 2:**
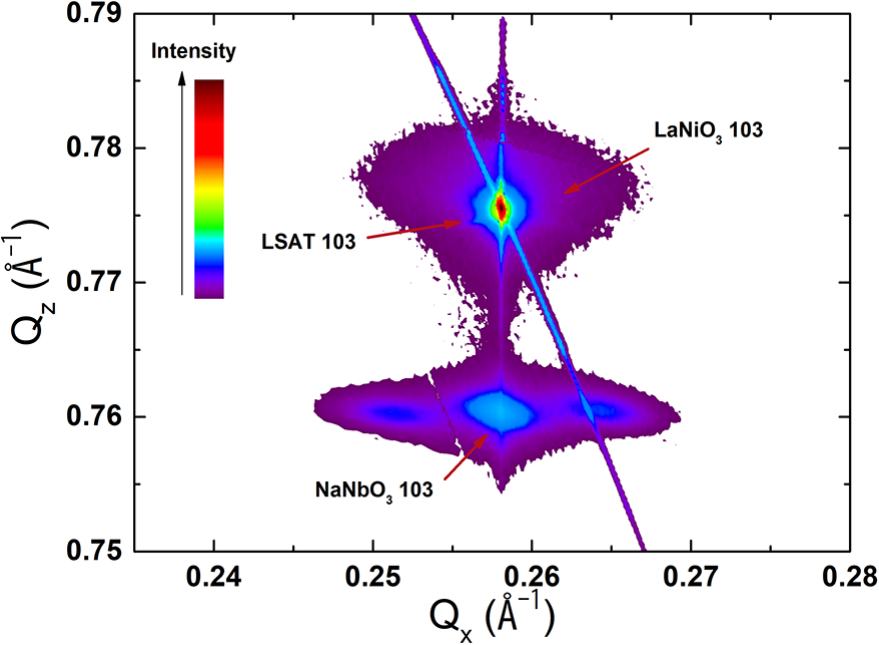
RSM of the 91 nm thick sample of NaNbO_3_ on LSAT.

Starting with the analysis of NaNbO_3_ films under compressive strain on LSAT (Figure 1a), the XRD patterns reveal the presence of two contributions from NaNbO_3_ already at a low thickness of 70 nm (NaNbO_3_ 004 and NaNbO_3_ 004*), both possessing a lattice parameter close to the expected value for a strained film. A third reflection (NaNbO_3_ 400) appears at thicknesses around 250 nm with a lattice parameter close to the bulk lattice parameter of NaNbO_3_ (*a*_PC_). Hence, this contribution can be attributed to a relaxation phenomenon. For a clear determination of whether the appearance of the additional reflections is due to relaxation of the film or other effects, reciprocal space maps (RSM) were recorded around the 103 substrate reflection (see the Supporting Information). For growth on LSAT, these measurements confirm the hypothesized partial relaxation at thicknesses of 250 nm and above, while the secondary contribution at lower thicknesses (NaNbO_3_ 004*) appears to be fully strained. Satellite peaks with different *Q*_*x*_ but the same *Q*_*z*_ value as the NaNbO_3_ film peak are observed for the films with thicknesses of 91 and 105 nm; Figure 2 shows the 91 nm thick sample as an example. These reflections indicate the presence of an in-plane periodic modulation with a periodicity of ≈15.5 nm. A prime candidate for the origin of this is a periodic modulation of the out-of-plane polarization component, which has been observed before for PbTiO_3_ thin films and heterostructures.^28,29^

The next substrate, SrTiO_3_, represents a special case, as visible from Table 1: depending on the orientation, the film can grow under compressive as well as tensile strain. Similar to the growth of NaNbO_3_ on LSAT, going beyond a critical thickness leads to the appearance of another reflection visible in the XRD pattern. This occurs for growth on SrTiO_3_ at around 130 nm. Calculation of the corresponding out-of-plane lattice parameters reveals the association to different growth orientations of the NaNbO_3_ thin films: the initial orientation features a lattice parameter matching the *b*/*c*-orientation, while the second reflection matches the *a*-orientation. However, unlike growth on LSAT, the RSMs confirm that both contributions are fully strained, even up to the 310 nm thick film. Thus, a unique strain relief mechanism can be observed, in which NaNbO_3_ changes growth orientation instead of relaxing to the bulk structure. This minimizes the overall strain, which is induced into the film and allows to grow thicker films without a relaxation effect. For a more detailed discussion of this film–substrate combination, see our previous publication.^2^

The growth of NaNbO_3_ under tensile strain, on DyScO_3_ and GdScO_3_, reveals the expected single contribution for thin films (NaNbO_3_ 400) until relaxation occurs and a second contribution with a bulk-like lattice parameter appears (NaNbO_3_ 400*). This relaxation, also confirmed by RSM (see the Supporting Information), appears for growth on DyScO_3_ at a thickness of around 90 nm and for GdScO_3_ between 22 and 70 nm. As growth on GdScO_3_ leads to larger strain, the relaxation starts at lower thicknesses. The out-of-plane lattice parameter of the strained phase is in both cases larger than that of the relaxed film, whereas, due to the tensile strain, a lowering of the out-of-plane lattice parameter is expected. Additionally, both cases show a decrease of the out-of-plane lattice parameter with film thickness until relaxation occurs. For further thickness increase, this contribution remains at a constant lattice parameter. It can be assumed, that the increasing strain in the film shifts the out-of-plane lattice parameter to lower values, which is in accordance with the expected effect of tensile strain on the out-of-plane lattice parameter. Effects that can lead to an increase of the lattice parameter could be related to defects, i.e., vacancies or cation intermixing, and off-stoichiometry.^30^ The latter can be excluded, as the film stoichiometry was previously optimized via X-ray photoelectron spectroscopy (XPS), and afterward, synthesis conditions were kept constant.^2^ Hence, one possible reason for the reduced out-of-plane lattice parameter is defect formation, which has been shown to be able to shift reflections in XRD by several degrees.^30,31^ This observation agrees with the fact, that NaNbO_3_ is prone to defect formation, especially Na and O vacancies.^32^ As the oxygen content was not analyzed for the films due to exposure to air before the XPS measurements, oxygen deficiency could contribute to the unexpectedly large increase of the lattice parameter. While this has not been observed for the growth on SrTiO_3_, oxygen deficiency might also be the cause for the peak splitting, which is observed for growth on LSAT (NaNbO_3_ 004 and NaNbO_3_ 004*). A possible reason as to why this does not occur for growth on SrTiO_3_ could be that, of the selected substrates, only SrTiO_3_ has a tendency to give away oxygen ions, which can then compensate the oxygen deficiency in the NaNbO_3_ during film growth. DyScO_3_ and GdScO_3_ have been shown to be stable against oxygen loss.^33^

**Figure 3:**
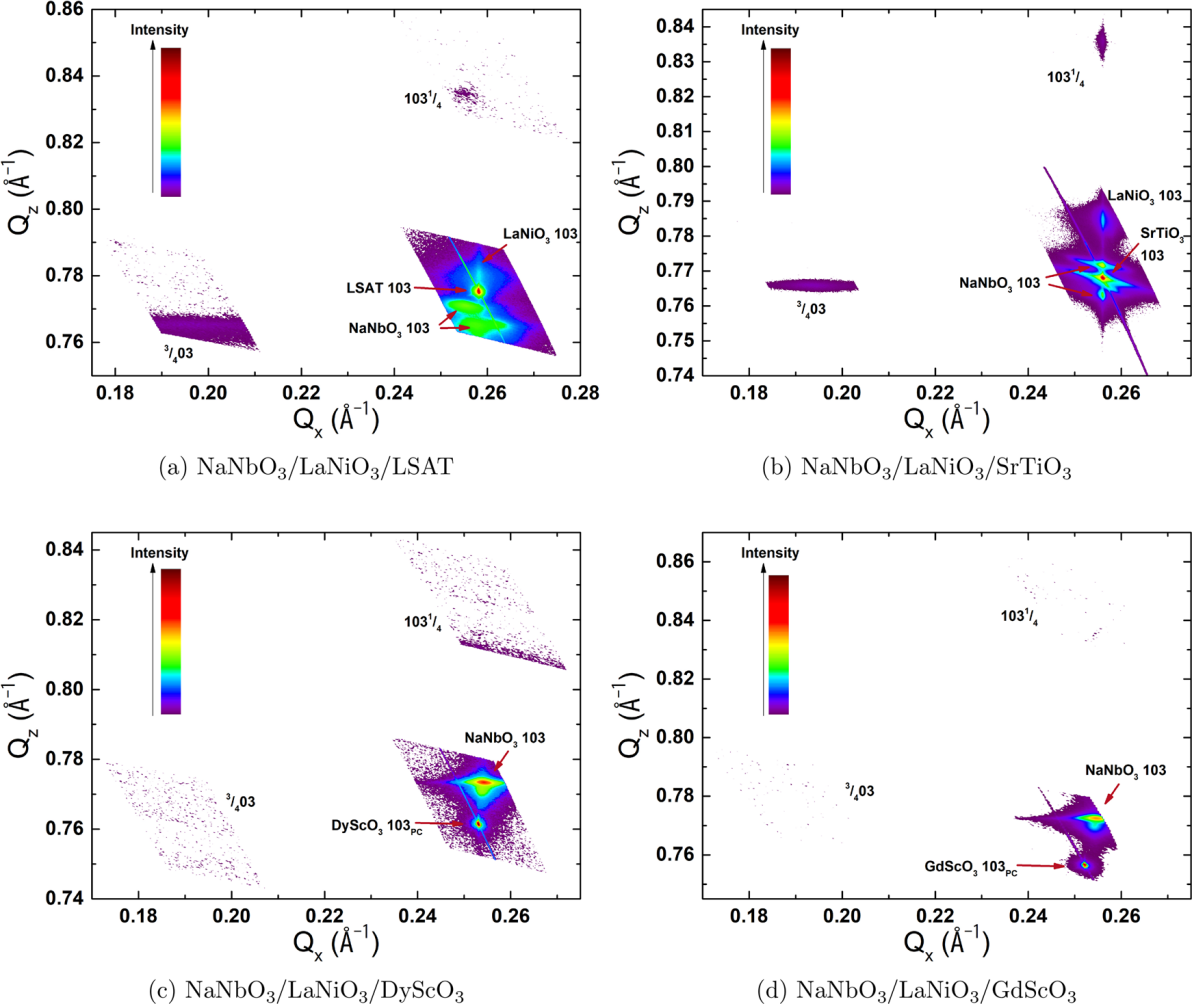
RSMs of NaNbO_3_ thin films grown on different substrates. The mapped region is around the 103 reflection of the substrates. Additionally, the areas for the superlattice reflections in-plane, , and out-of-plane, , are shown.

The difference in the crystal structure of the AFE and FE phases of NaNbO_3_ can be utilized as a criterion to identify the ground state of NaNbO_3_.^34,35^ This has been applied to our thin films, via measurement of the expected area for the  superlattice peaks in the reciprocal space. The results for the thickest films on each substrate are shown in Figure 3.

The presence of a  superlattice reflection can only be identified on two of the four substrates, LSAT and SrTiO_3_, while no intensity is detected in the measured regions in the case of tensile strain, on DyScO_3_ and GdScO_3_. This indicates the presence of a bulk like AFE ground state for the case of compressive or small tensile strain, while larger tensile strain appears to inhibit the formation of this phase completely. This effect seems to go beyond the relaxation to the bulk lattice parameters, as the shown RSMs were measured on the thickest films, i.e., films that are already relaxed. In the case of compressive strain on LSAT, the relaxed fraction also shows a superlattice peak, as visible in Figure 3a. Here, the in-plane superlattice peak, , is very clear and originates from the strained fraction of the film, as evidenced by the same *Q*_*z*_ value as the strained NaNbO_3_ film. Meanwhile, the peak with very low intensity in the out-of-plane direction,  belongs to the relaxed fraction, as both show the same *Q*_*x*_ value. This perfectly matches the expected direction for the cell quadrupling based on the bulk AFE lattice:^36^ for the strained film, the *a*_PC_ lattice parameter is expected to grow in-plane to minimize the total strain. Meanwhile the out-of-plane lattice parameter of the relaxed fraction coincides with the *a*_PC_ lattice parameter (see Figure 1a), and hence, the superlattice peak should appear out-of-plane. The same relation can be found for the growth on SrTiO_3_, where the superlattice peak positions also match the expected directions of the bulk AFE structure,^2^ further confirming the close correlation with the bulk structure of NaNbO_3_. Notice that, for the orientation growing with small tensile strain on SrTiO_3_, the superlattice peak appears as well. On the contrary, for both the RSM on DyScO_3_ (Figure 3c) and GdScO_3_ (Figure 3d), no intensity past some background noise can be detected in the regions where the superlattice peaks are supposed to appear. This is unexpected, as the films shown here are mostly relaxed to bulk structure. It seems that the epitaxial relation on substrates generating tensile strain destabilizes the formation of the bulk AFE phase even above the relaxation thickness.

**Figure 4:**
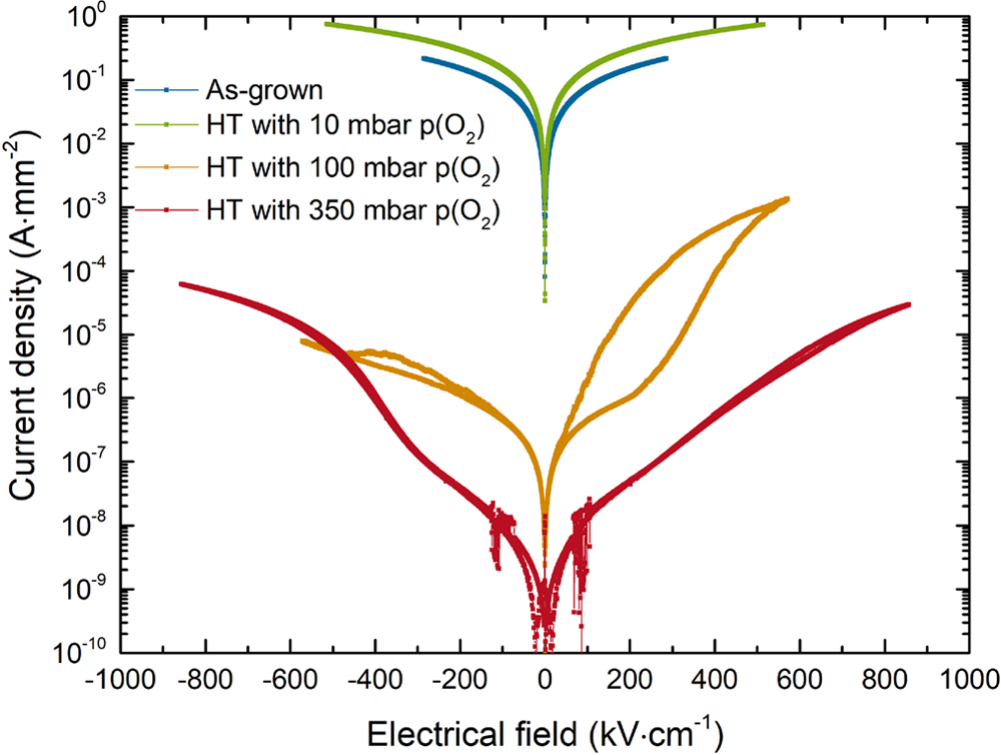
Leakage current of NaNbO_3_ thin films grown on SrTiO_3_ for an as-grown sample as well as samples after heat treatment (HT) with different oxygen pressures during the annealing step.

For further analysis of the phase balance between the AFE and FE phases, electrical measurements were conducted to evaluate the polarization versus electric field behavior. Initial measurements revealed a large leakage current. It was hypothesized that the origin of this leakage current was related to oxygen vacancies, as is often the case in perovskite thin films,^25,30,37^ and has already been discussed as a possible origin to the peak shifts observed in the XRD scans. To validate this hypothesis, a series of heat treatments under varying oxygen pressures from 10 to 350 mbar was carried out on NaNbO_3_ thin films grown on SrTiO_3_(001) under the same conditions. For all heat treatments, the temperature was fixed at 400 °C for 1 h. The results show a clear reduction of the leakage current with increasing oxygen pressure during the heat treatment (see Figure 4). Annealing at low oxygen pressures increases the leakage compared to the as-grown sample, most likely due to the formation of additional oxygen vacancies due to the too low oxygen pressure or to sodium loss in the sample at the higher temperature.

The measured data was fitted with regard to possible leakage mechanisms in thin films (see the Supporting Information).^38^ For the case of the films with high leakage current, i.e., as-grown and annealed with 10 mbar of oxygen, the leakage over the whole measurement range can be attributed to Ohmic conduction. Similarly, the fittings of the films annealed with 100 and 350 mbar show Ohmic conduction being the main mechanism at lower fields with a transition to another mechanism at around 250 kV·cm^–1^, with slight variation of the transition field for positive and negative bias. For the thin film annealed at 100 mbar, the Poole-Frenkel mechanism seems to be present at large positive electric fields. For thin films annealed with 350 mbar, with reduced leakage current, the best fitting result is achieved for the Schottky mechanism. In both cases, the leakage current in the negative polarity follows another mechanism. This difference of mechanism can be attributed to the different materials used for the top and bottom electrodes, hinting at an electrode controlled leakage current mechanism. This mechanism in the negative polarity is related to a space charge limited current (SCLC), indicating lower energy barriers for carrier injection in this direction.^38^

While all films show Ohmic conduction at low fields, the magnitude of this current severely decreases with increasing pressure during annealing, indicating a reduction of charge carriers.^39^ Similarly, while Poole–Frenkel conduction appears for the film annealed with 100 mbar, originating from defect states in the band gap, the film annealed with 350 mbar shows in both directions electrode controlled leakage current mechanisms, revealing the reduction of defects in the film. The origin of the high leakage for untreated films can thus be related to the presence of oxygen vacancies. A possible mechanism leading to the formation of oxygen vacancies during film growth could be related to an intermixing of the Na and Nb cations, which has been observed for similar perovskites.^40^

Structural characterization of the samples before and after heat treatment revealed neither a change in the crystal structure nor in the strain state (see the Supporting Information).

**Figure 5:**
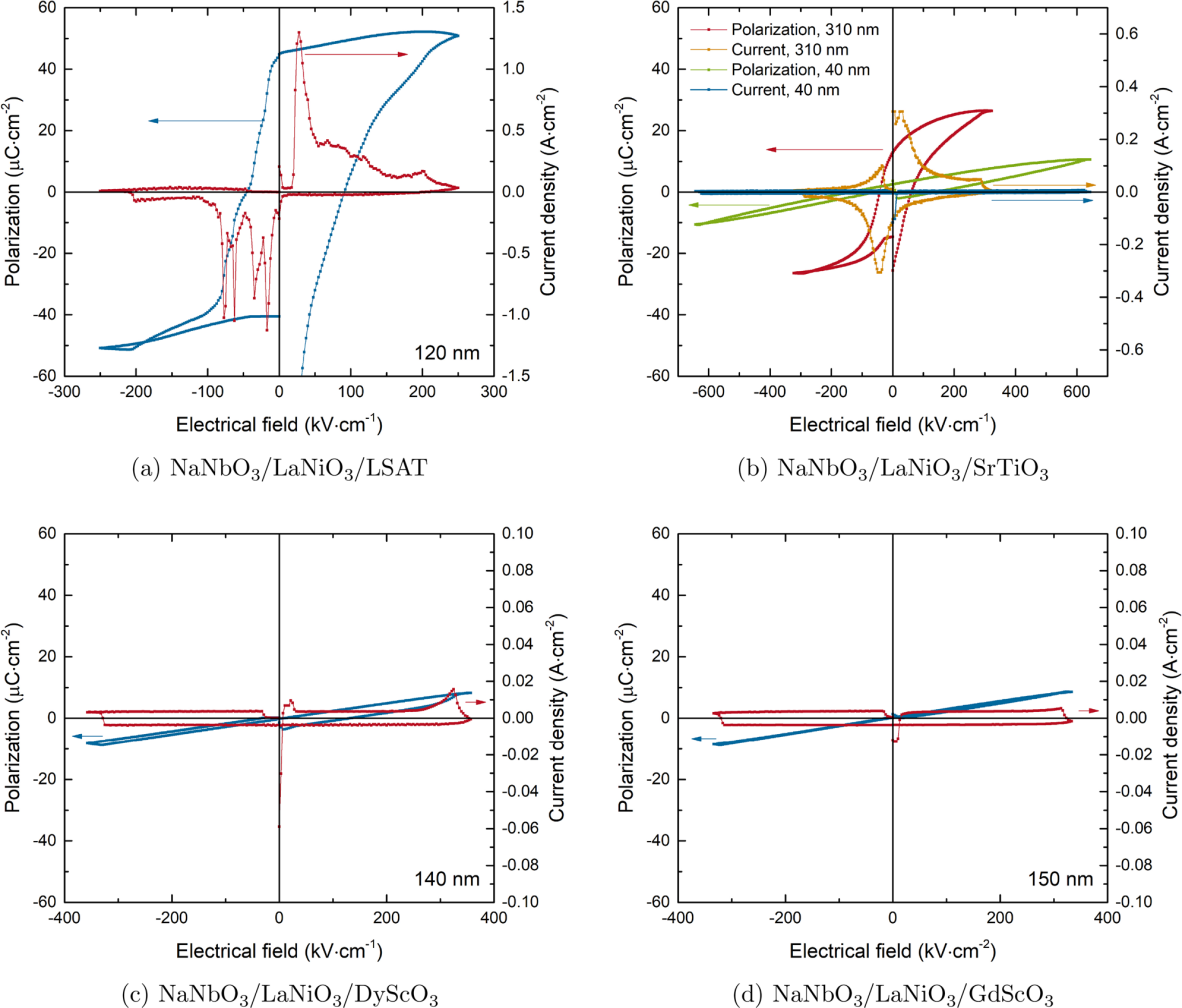
Polarization versus electric field results for NaNbO_3_ thin films. Only the films with the lowest leakage currents are shown for LSAT, DyScO_3_, and GdScO_3_ substrates, representing the behavior of all films on these substrates, with their respective thickness indicated in the graph. The right axis shows the current density depending on the applied field.

After the decrease of the leakage current by the heat treatment, further electrical characterization of the samples could be conducted. As the polarization vector for the bulk ground state lies in the [110]_PC_ direction, measuring the out-of-plane contribution with the parallel plate capacitor setup will give access to a part of the polarization for the case of epitaxial growth on the selected substrates.^41^ To complement the information about the phase balance from XRD, the polarization versus electric field behavior was measured, as shown in Figure 5. For each substrate, the curves shown represent the films with the lowest leakage current. The NaNbO_3_ thin films show a different electrical behavior depending on the type of epitaxial strain: in the case of compressive strain, on LSAT (Figure 5a), it is apparent from the saturation polarization of up to 55 μC·cm^–2^ that the film exhibits a strong out-of-plane polarization component. The magnitude of this response is more than twice as large as the saturation polarization for the growth on SrTiO_3_, confirming the effect of compressive strain on the polarization.^9,42^ This measured saturation polarization is also larger than the highest values measured for NaNbO_3_ bulk materials of ≈40 μC·cm^–2^.^8^ It should be noted that thickness impacts the dielectric properties of thin films, affecting the comparison between these films on different substrates. However, due to the dead layer effect, thicker films tend to have higher permittivity and polarization values; therefore, the mentioned magnitude of increase in the saturation polarization should not be negatively affected.^43^ Meanwhile, as visible from the shape of the polarization versus electrical field hysteresis resulting from multiple peaks in the plot of the current density versus electrical field, no clear conclusion can be drawn on the type of dielectric for this case. In combination with the results from the structural characterization, a possible reason for this response could be the presence of complex domain ordering^28^ or the overlapping of different contributions. For growth on SrTiO_3_, no polarization contribution becomes visible up to the thickest film (Figure 5b). The latter exhibits bulk like properties, while for the thinner films, the transition from the antipolar phase, as detected in the RSMs, to a FE phase is not achieved, indicating that the transition is inhibited by epitaxy. However, whether the main impact of strain is an inhibition of the transition or a manipulation of the phase balance for the AFE is not clear based on these results. Thus, for thin films grown under compressive strain up to small tensile strain, no clear impact of the strain on the phase balance can be determined and a bulk like behavior with a field-induced phase transition at low fields can be observed. For the case of tensile strained-grown NaNbO_3_ on DyScO_3_ and GdScO_3_, only a linear behavior can be detected in the polarization versus electric field measurement until the leakage current contributes at higher applied voltages (Figure 5c,d). As the structural analysis revealed no antipolar displacements for this case, the stabilization of such a phase can be excluded for these films. Therefore, the most likely explanations are either that a different structure is formed for the growth on these substrates or that the polarization direction is rotated fully in-plane due to the tensile strain.^26,44,45^ The latter would result in a linear behavior, as the measurement with the utilized parallel-plate electrode setup only measures the out-of-plane contribution. In order to clearly differentiate these two cases, in-plane measurements would be necessary.

## 4 Summary

The strain impact on NaNbO_3_ thin films has been thoroughly investigated in terms of structural and electrical properties. The epitaxial strain has been varied from compressive to tensile via epitaxial growth on four different substrates by XRD (see Figure 1). The structural characterization revealed the formation of a bulk-like AFE phase as ground state for thin films from a compressive strain of 0.8% to a small tensile strain of −0.2%, while larger tensile strain completely inhibited the formation of this phase, even after relaxation.

**Figure 6:**
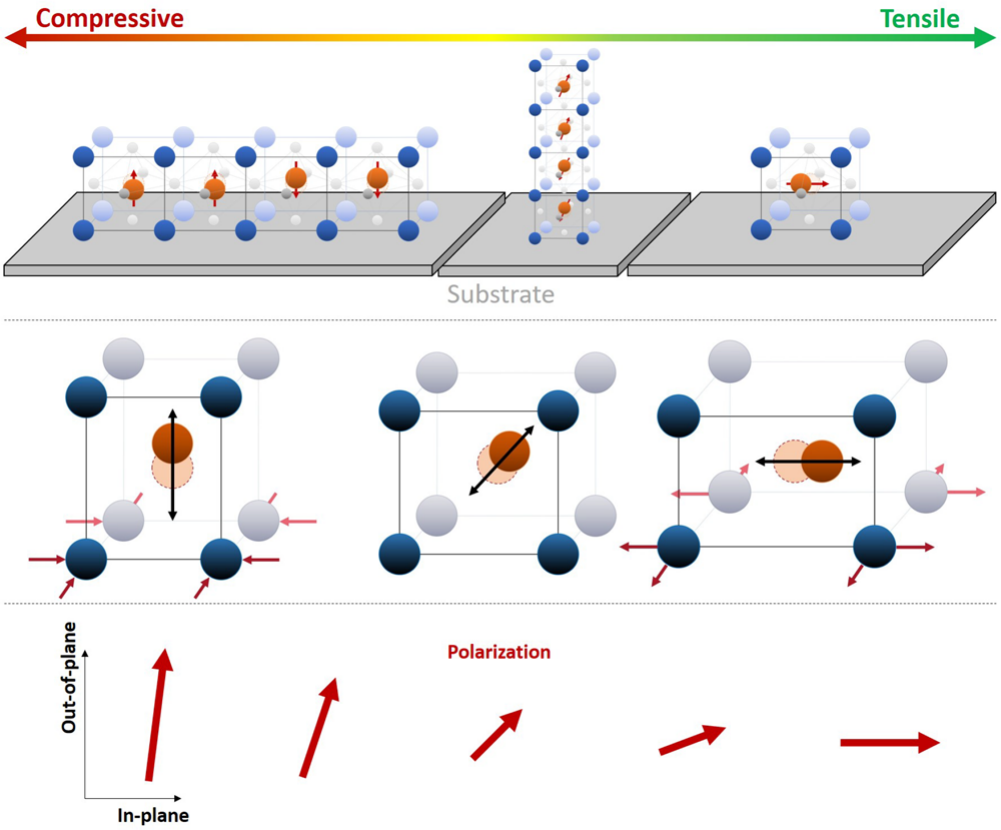
Overview of the evolution of properties from compressive to tensile strain. Top: Schematic of the growth direction of the NaNbO_3_ based on the unit cell multiplicity identified from RSM. Center: Simplified schematic representation of a unit cell with a displaced ion in the center under the different strain states. Bottom: Projection of the polarization vector for the evolution from compressive to tensile strain.

Correlation of the structural and electrical results reveals a more complicated picture, with the exact type of dielectric being unclear for the film under compressive strain and the appearance of a FE hysteresis for small tensile or compressive strain. Meanwhile, no polarization was measured for the case of larger tensile strain, indicating a potential rotation of the polarization fully into the in-plane direction (see Figure 6). While no stabilization of the AFE structure with compressive nor tensile strain can be confirmed, the retention the AFE ground state by application of compressive strain along with the increase in saturation polarization shows the potential of strain engineering for AFE materials. With regard to the application for energy storage, this promises a large increase in the recoverable energy density. With suitable doping, which is already deeply investigated for bulk NaNbO_3_,^6,18^ it is expected that the AFE phase can be further stabilized for thin films. Then, the increase of the polarization translates directly to larger energy densities, showing a promising route for the future of AFE materials.

## Data Availability

The data that support the findings of this study are available from the corresponding author upon reasonable request.

## Supporting Information Available

Discussions of detailed information on the RSMs indicating the strain state for each film, with calculation of intensity ratios of the superlattice peak for estimation of the magnitude of the ion displacements, further details concerning the leakage current analysis, and evaluation of the impact of the heat treatment on structural properties and figures of RSMs recorded for the thickness series, extracted line shapes from the RSM, leakage current fittings, and comparisons of the structural results before and after the heat treatment (PDF)
