# Supplementary material for: Impact of Strain Engineering on Antiferroelectricity in NaNbO3 Thin Films
Source: ACS Omega. 2023 Jun 20;8(26):23587–95. doi: 10.1021/acsomega.3c01327 (PMC10324056; doi:10.1021/acsomega.3c01327)
Supplement: Supplementary file 1 — ao3c01327_si_001.pdf [file ao3c01327_si_001.pdf]

Impact of Strain Engineering on Antiferroelectricity in $NaNbO_3$ thin films

Thorsten Schneider,*,† Juliette Cardoletti,†,‡ Philipp Komissinskiy,† and Lambert Alff†

†*Institute of Materials Science, Technische Universität Darmstadt, Alarich-Weiss-Strasse 2, 64287 Darmstadt, Germany*

‡*Now at: Materials Research and Technology Department, Luxembourg Institute of Science and Technology (LIST), 41 rue du Brill, 4422 Belvaux, Luxembourg*

E-mail: thorsten.schneider@tu-darmstadt.de

Supplementary Information

The supplementary information includes the discussion on the reciprocal space maps (RSM) concerning the strain state of the thin films, the fitting of the superlattice reflections to compare the magnitude of the antipolar displacements as well as the analysis of the leakage current for the effect of the heat treatment on $NaNbO_3$ thin films.

Reciprocal space maps

All thin films have been characterized via RSMs to determine their strain state. These are displayed in Figures S1, S2, S3 and S4 for growth on LSAT, $SrTiO_3$, $DyScO_3$ and $GdScO_3$, respectively. For each RSM, the area around the 103 substrate reflection was chosen to evaluate in-plane as well as out-of-plane contributions.

For growth on LSAT (Figure S1), the reflection of the $LaNiO_3$ bottom electrode is mostly hidden by the substrate reflection, while the reflections for $NaNbO_3$ are at smaller Q_z values, as visible in the graphs. The first thin film that shows a clear contribution from a relaxed phase is the film with a thickness of 240 nm, whereas the film with 120 nm reveals a peak splitting which is not related to a relaxation, since both contributions feature the same Q_x as the substrate reflection. This effect, however, is not visible for thin films with lower thickness. Thin films of 91 nm and 106 nm thickness show additional peaks as satellites to the $NaNbO_3$ main reflection in Q_x direction, as discussed in the main part of this publication.

For growth on $SrTiO_3$ (Figure S2), thin films with low thickness have most of their $NaNbO_3$ reflection hidden in the substrate reflection, however, some intensity of their reflection is visible towards lower values of Q_z. At a thickness of 130 nm, the second contribution towards larger values of Q_z starts to become visible, growing clearer with further increase in thickness. With this, it also becomes evident, that all contributions from the $NaNbO_3$ grow fully strained, without any relaxation.[1]

For growth on $DyScO_3$ (Figure S3) and $GdScO_3$ (Figure S4), the structural behavior is similar, with the exception of the onset of relaxation, which is occurring at lower thicknesses for growhth on $GdScO_3$ due to the larger strain. While a clear change of the peak position is recognizable upon appearance of the second contribution from $NaNbO_3$ as identified by the θ-2θ scans (see Figure 1c and d), already the first reflection is broadened towards larger Q_x. This transition is found to occur between 68 nm to 96 nm for $DyScO_3$ and between 12 nm to 22 nm for $GdScO_3$. For the latter, the second contribution becomes only clearly discernible at 93 nm, however.

The superlattice peaks of the 400 nm thick $NaNbO_3$ on LSAT were fitted to extract the peak area and estimate the antipolar displacement by the intensity of the superlattice peak.[1] The extracted line shapes as well as the fittings are shown in Figure S5.

The fitting of the out-of-plane superlattice peak (Figure S5b) with the corresponding film peak of the RSM (Figure S5a) results in an intensity ratio of 0.26 % while the same fitting for the in-plane direction (Figure S5c and d) results in a ratio of 6.3 %. Notice that for the out-of-plane film peak (Figure S5a), the extracted line scan shows not only the $NaNbO_3$ reflection ($'Peak1'$), but also the $LaNiO_3$ ($'Peak3'$) and LSAT ($'Peak2'$) reflections. For the in-plane case, both fitted peaks belong to $NaNbO_3$, with one corresponding to the strained ($'Peak2'$) and one to the relaxed fraction ($'Peak1'$). As the total intensity of the superlattice peak is not very high, the fitting error cannot be neglected in the evaluation, as is especially clear for the in-plane superlattice peak (Figure S5d).

The resulting intensity ratios for $NaNbO_3$ thin films on $SrTiO_3$ are 1.7 % and 6.7 %, out-of-plane and in-plane, respectively.[1] Thus, comparison of the intensity ratios for the growth of $NaNbO_3$ on different substrates reveals similar values given the uncertainty of the fitting and, hence, allows no clear conclusion whether the compressive strain induced by LSAT leads to further stabilization of the AFE state.

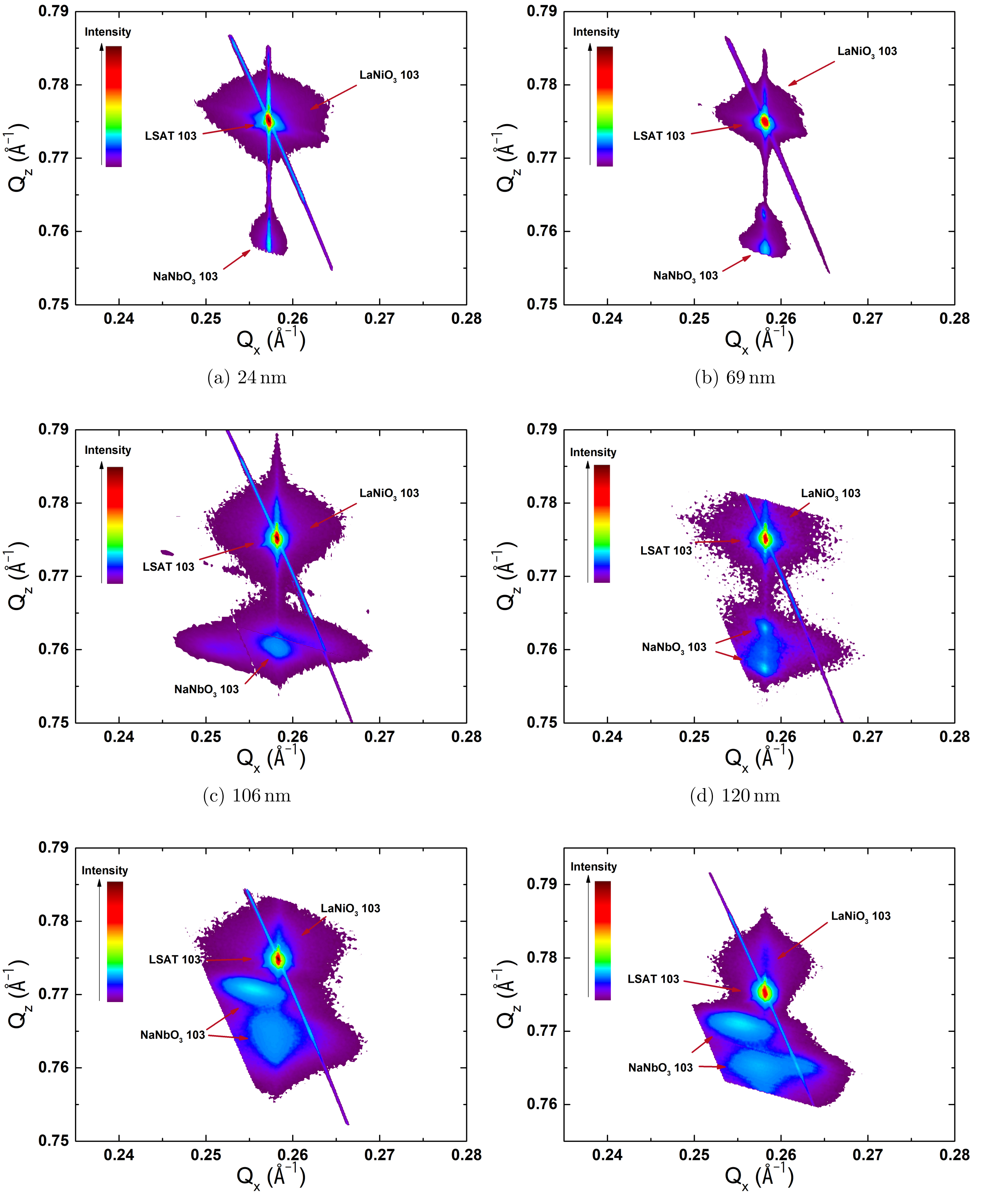

(a) 24 nm (b) 69 nm

(c) 106 nm (d) 120 nm

(e) 240 nm (f) 400 nm

Figure S1: RSMs recorded for the thickness series of $NaNbO_3$ grown on LSAT with $LaNiO_3$ as the bottom electrode.

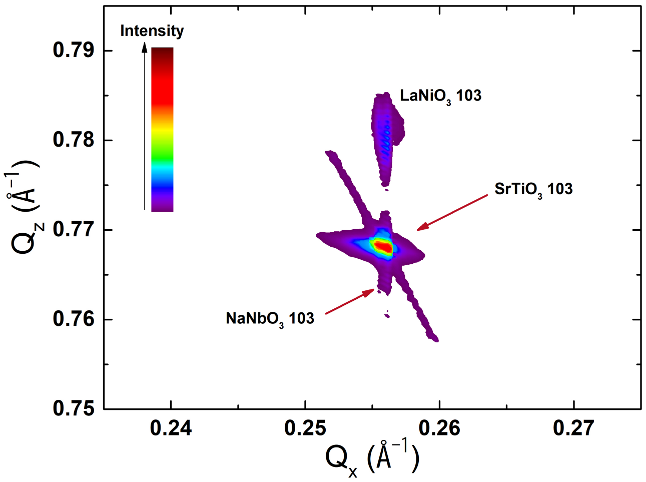

(a) 10 nm

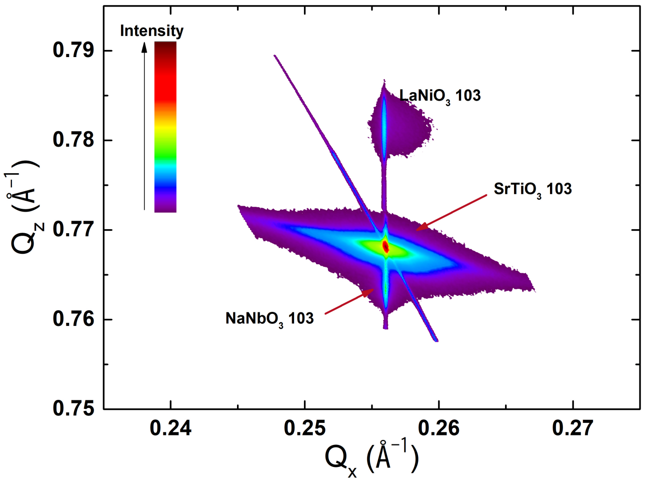

(b) 40 nm

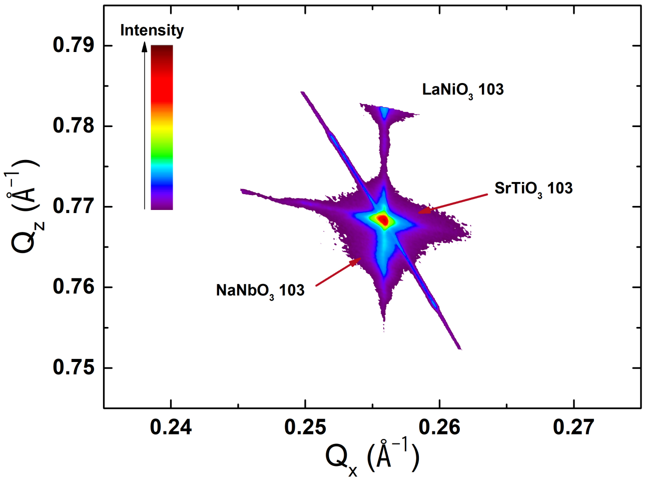

(c) 50 nm

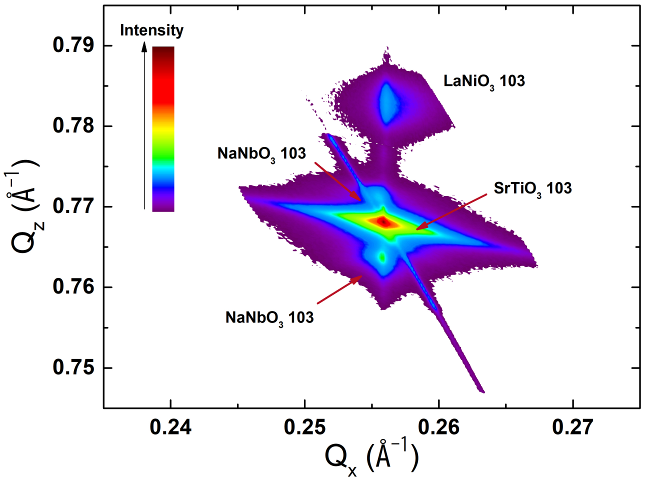

(d) 130 nm

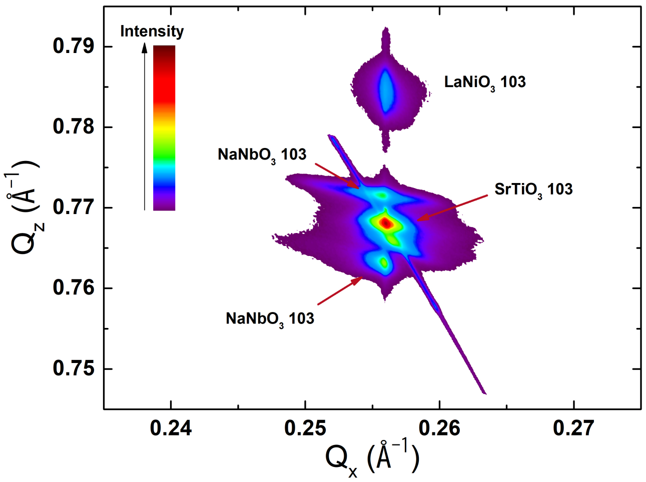

(e) 200 nm

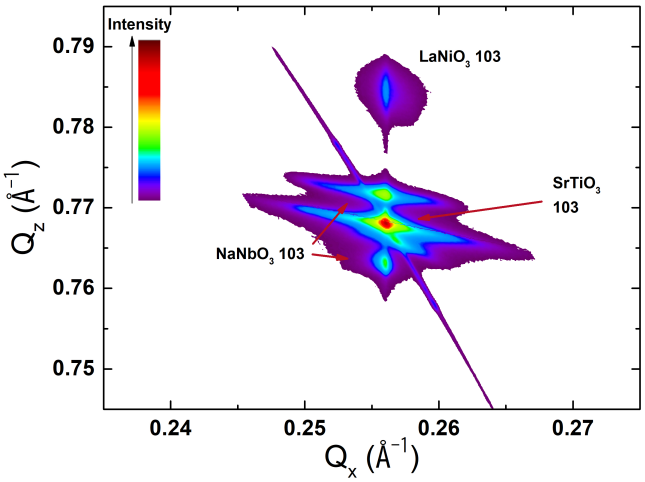

(f) 310 nm

Figure S2: RSMs recorded for the thickness series of $NaNbO_3$ grown on $SrTiO_3$ with $LaNiO_3$ as the bottom electrode.

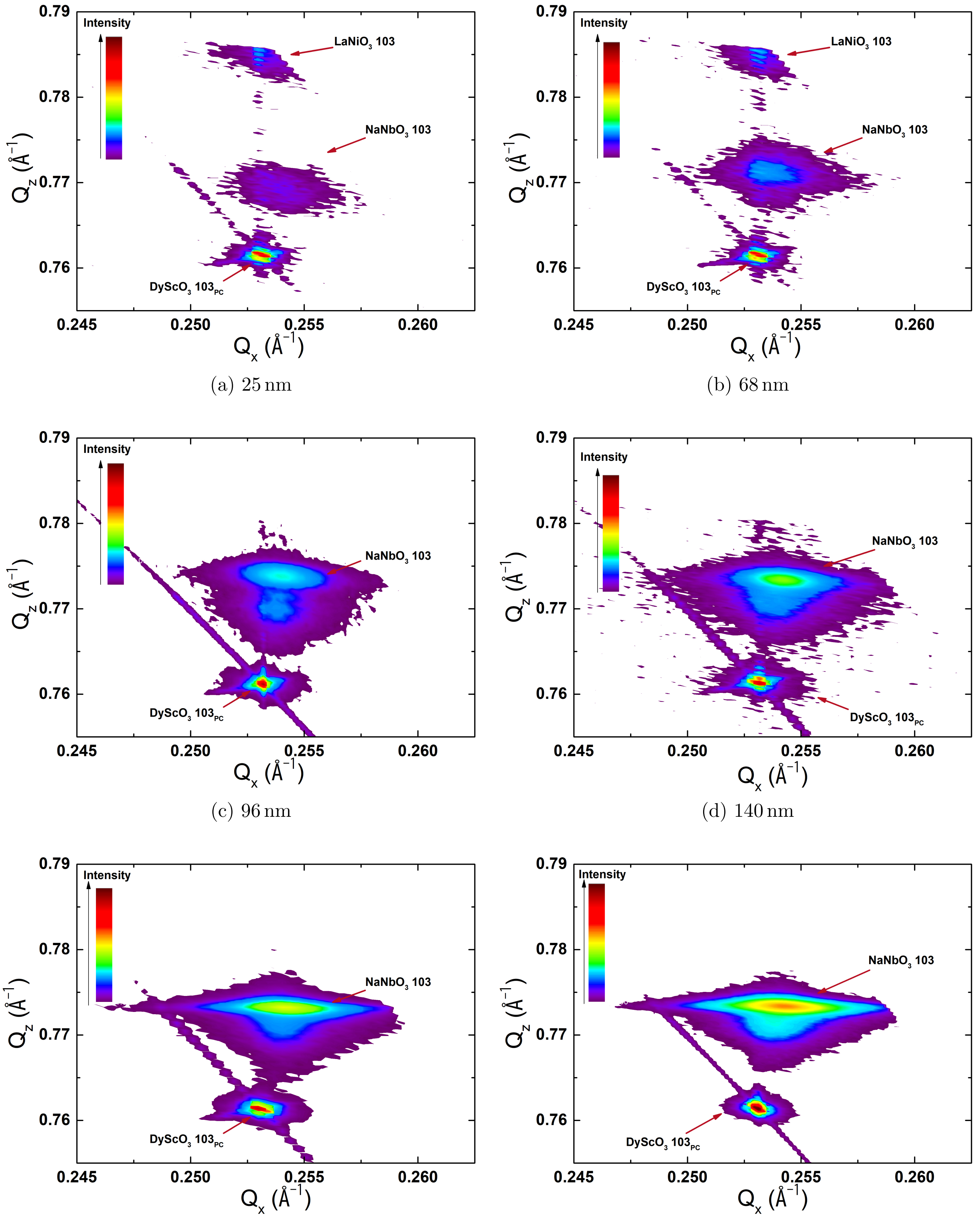

(a) 25 nm

(b) 68 nm

(c) 96 nm

(d) 140 nm

(e) 280 nm

(f) 400 nm

Figure S3: RSMs recorded for the thickness series of $NaNbO_3$ grown on $DyScO_3$ with $LaNiO_3$ as the bottom electrode.

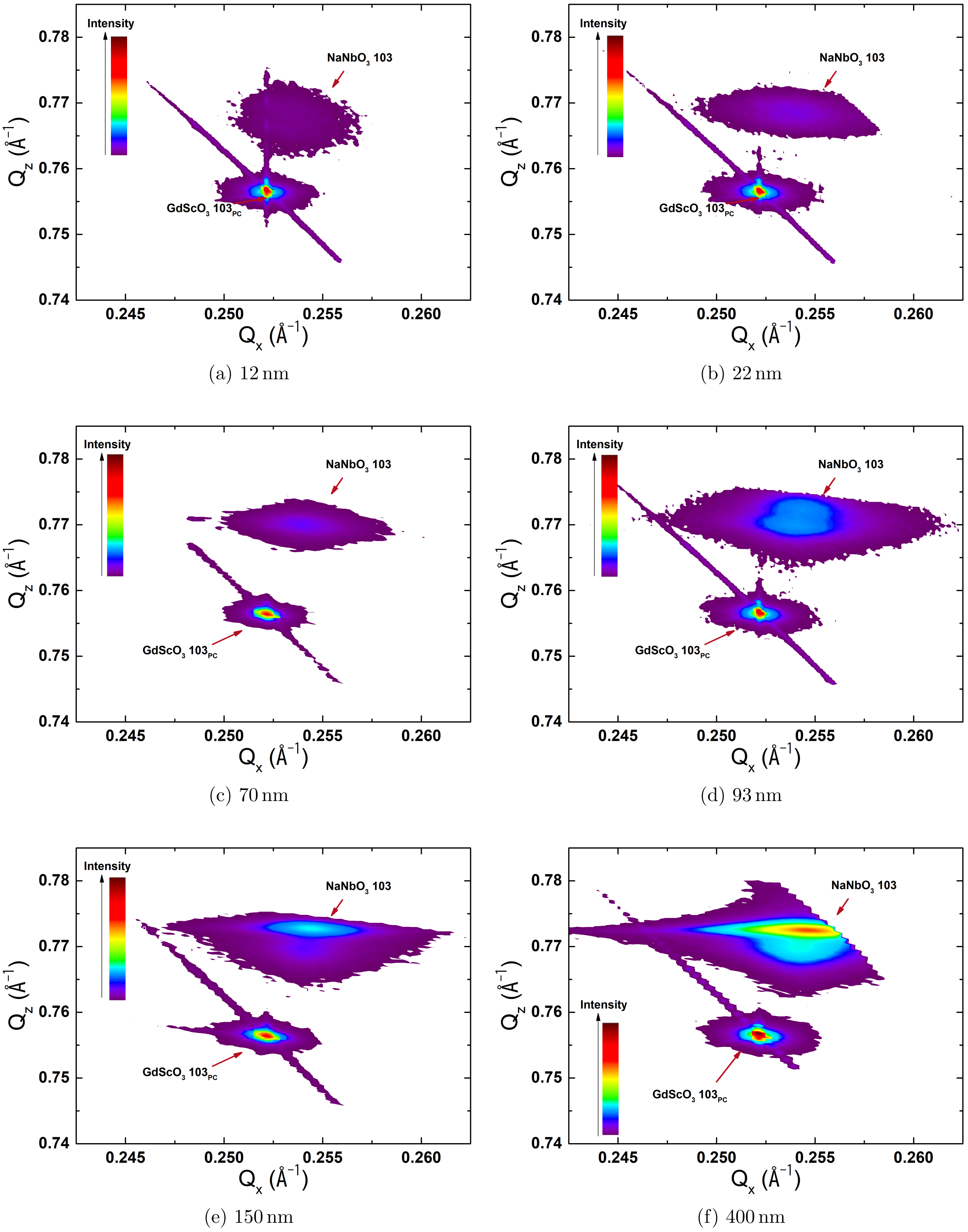

(a) 12 nm (b) 22 nm (c) 70 nm (d) 93 nm (e) 150 nm (f) 400 nm

Figure S4: RSMs recorded for the thickness series of $NaNbO_3$ grown on $GdScO_3$ with $LaNiO_3$ as the bottom electrode.

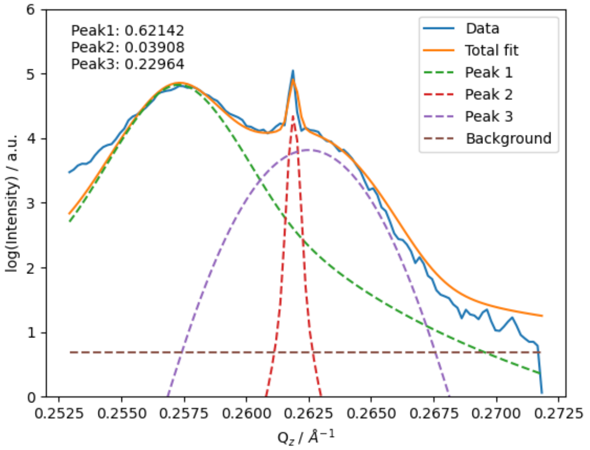

(a) Extracted line from the RSM around the 103 substrate reflection at $Q_x = 0.255$. $'Peak1'$, $'Peak2'$ and $'Peak3'$ denote the $NaNbO_3$ 103, LSAT 103 and $LaNiO_3$ 103 reflection, respectively.

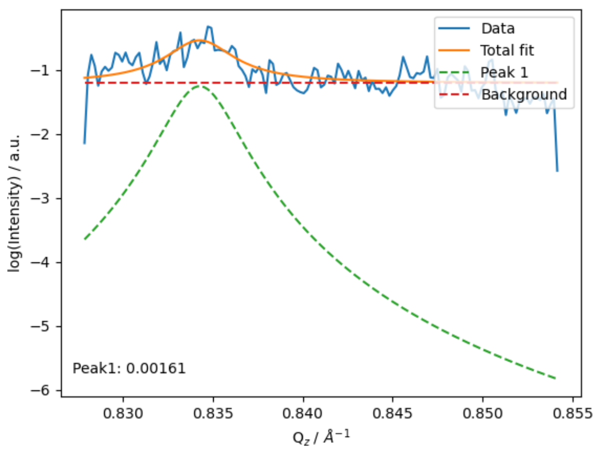

(b) Extracted line from the RSM for the $103\frac{1}{4}$ superlattice peak at $Q_x = 0.255$. $'Peak1'$ denotes the $NaNbO_3$ $103\frac{1}{4}$ reflection.

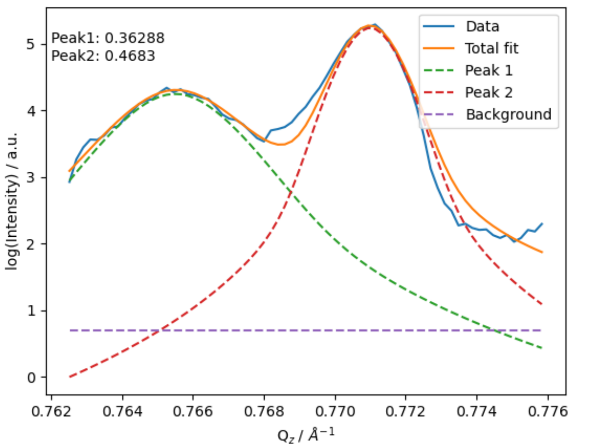

(c) Extracted line from the RSM around the 103 substrate reflection at $Q_z = 0.765$. $'Peak1'$ and $'Peak2'$ both denote the $NaNbO_3$ 103 reflection, with the former representing the strained and the latter the relaxed fraction.

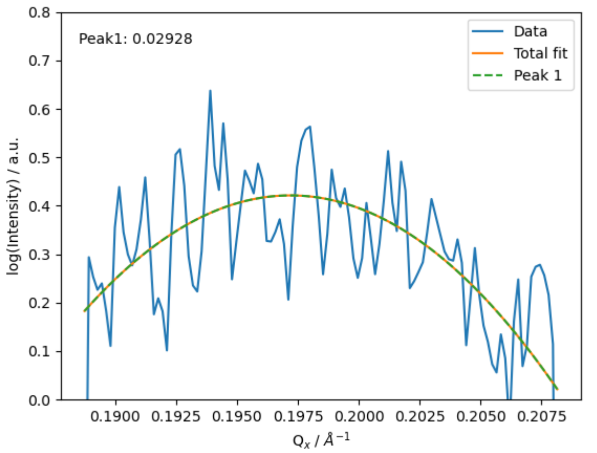

(d) Extracted line from the RSM for the $\frac{3}{4}03$ superlattice peak at $Q_z = 0.765$. $'Peak1'$ denotes the $NaNbO_3$ $\frac{3}{4}03$ reflection.

Figure S5: Extracted line shapes from the RSM of the 400 nm thick $NaNbO_3$ film on LSAT which shows fittings for the different film reflections. Included in the graphs are the areas calculated for the different peaks in the top left corner (for (b) in the bottom left corner).

Leakage current

The leakage current curves shown in Figure 3 have been fitted to investigate the mechanisms[2,3] responsible for the leakage current.
The considered mechanisms were Ohmic conduction, space charge limited current (SCLC), Poole-Frenkel and Schottky conduction. Ohmic conduction is due to free charge carriers and a linear behavior with a slope of 1 is expected in a double logarithmic plot of the current density versus the electrical field, since $log(J) = log(E) + log(A)$, with J as the current density, E as the electric field and A a constant. SCLC shows a transition from Ohm's law to traps-filled limit current and Child's law, which both show a slope of 2 in the double logarithmic plot.[4] Poole-Frenkel conduction originates from the emission of charge carries from traps in the band gap and shows a linear behavior in the plot of $log(J/E)$ versus $E^{1/2}$ and from the slope the optical permittivity ϵ_{Op} can be calculated: $log(J/E) = \sqrt{\frac{q^3}{\pi\epsilon_0\epsilon_{Op}k_B^2T^2}} \cdot \sqrt{E} + A$ with q as the charge, ϵ_0 the vacuum permittivity, k_B the Boltzmann constant, T as the temperature and A a collection of various constants.[2] Lastly, Schottky conduction arises due to the injection of charge carriers from the electrode over the Schottky barrier. According to the formula $log(J) = \sqrt{\frac{q^3}{4\pi\epsilon_0\epsilon_{Op}k_B^2T^2}} \cdot \sqrt{E} + A$, a linear behavior should be visible for a plot of the logarithm of the current density versus the square root of the electrical field.[2] For the differentiation between Poole-Frenkel and Schottky, the optical permittivity ϵ_{Op} has been calculated from the slope of the respective fit. While no values for this permittivity have been determined for $NaNbO_3$ thin films, an assumption is based on the general assumption that $\sqrt{\epsilon_{Op}} = n$, n being the index of refraction, which is generally considered $n > 1$. Comparable perovskite compounds feature values for the refractive index around $2.2 - 2.5$,[5] hence the range expected for ϵ_{Op} is $\approx 4.8 - 6.3$.
For the as-grown film as well as the film annealed with 10 mbar of oxygen pressure, only the fitting corresponding to Ohmic conduction was carried out, as these matched the mechanism over the whole range (see Figures S6 and S7, respectively). Both films show a matching slope for the whole range of the applied electric field, indicating that Ohmic conduction due to free charge carriers is the dominating conduction mechanism. The deviation at small fields is due to measurement errors at low currents.
Analysis of the thin films annealed at 100 mbar and 350 mbar of oxygen pressure also shows the presence of Ohmic conduction at low fields, however, a change to a different mechanism can be observed by a change of the slope in the double-logarithmic plots (see Figures S8 and S9). This change appears at around $250\,\text{kV} \cdot \text{cm}^{-1}$, with slight variation for positive and negative bias, as marked in the graphs.
Looking at the thin film annealed with 100 mbar of oxygen pressure (Figure S8), a clear change of the slope can be recognized at a field of $\approx -100\,\text{kV} \cdot \text{cm}^{-1}$ and $\approx 220\,\text{kV} \cdot \text{cm}^{-1}$. For the negative polarity, the slope only increases slightly, indicating the presence of SCLC while for the positive side a much larger increase of the slope becomes visible. The fitting results from the Schottky and Poole-Frenkel mechanisms (shown in Figure S8c-f) reveal the clear absence of these mechanisms in the negative direction, while at high positive fields, the slope of the linear fit results in a optical permittivity of 5.02, which is in the expected range. Hence, it can be assumed, that this mechanism plays a role in the conductivity at large positive fields, possibly due to states in the band gap resulting from oxygen vacancies.[2]
Similarly, the film annealed with 350 mbar of oxygen pressure (Figure S9) shows a transition from the Ohmic behavior at larger electric fields. In both polarities, at first SCLC behavior is visible at intermediate fields, indicated in the double-logarithmic plots (Figure S9a and b), upon which another transition can be seen by a further increase of the slope. For the negative polarity, neither Schottky nor Poole-Frenkel reveal a good fit, however, the shape is similar to a transition from the traps-filled limit current to Child's law.[2] However, to validate this hypothesis, data points at larger fields would be necessary to reveal the slope of 2 in the double logarithmic plot. For the case of

positive polarity on the other hand, the slope of the Schottky fitting shows a optical permittivity in the expected range after the transition from SCLC at $\approx 270\,\mathrm{kV \cdot cm^{-1}}$. This confirms the reduction of the number of internal defects such that the conduction mechanism transforms from bulk controlled to electrode controlled and, hence, the success of the heat treatment.

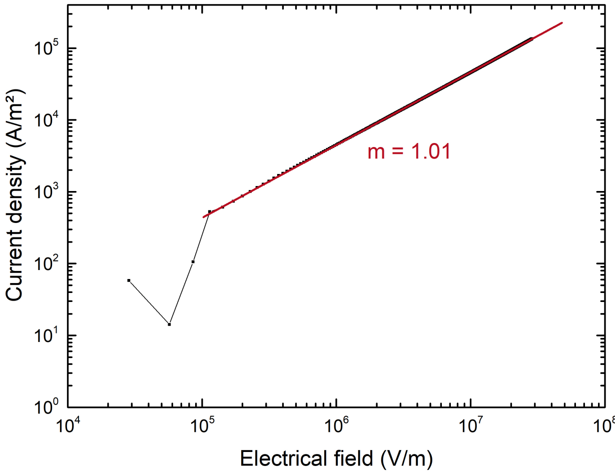

(a) Negative polarity, Ohm fitting

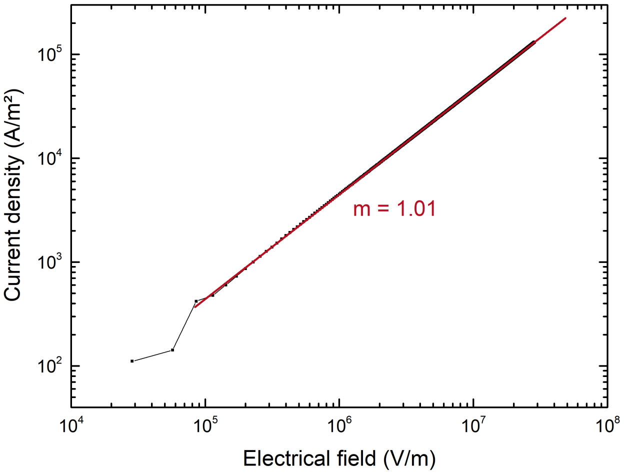

(b) Positive polarity, Ohm fitting

Figure S6: Leakage current fittings for the $NaNbO_3$ thin film as-grown.

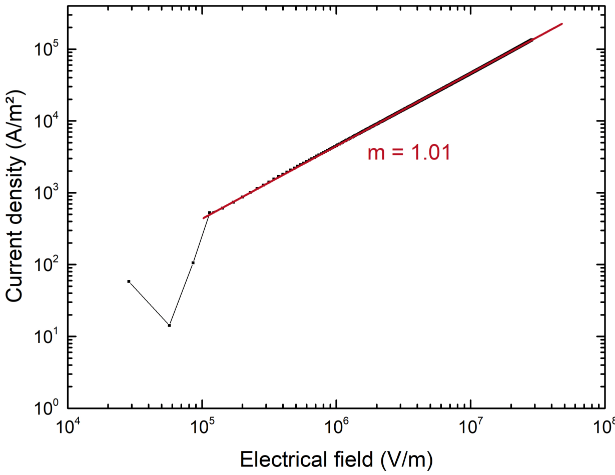

(a) Negative polarity, Ohm fitting

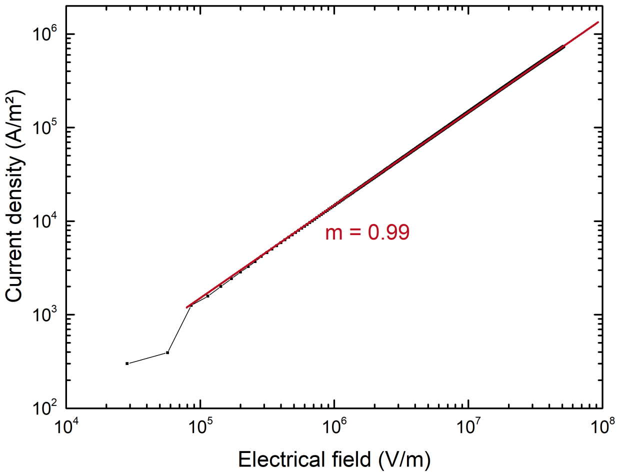

(b) Positive polarity, Ohm fitting

Figure S7: Leakage current fittings for the $NaNbO_3$ thin film annealed with $p(O_2) = 10\,\mathrm{mbar}$.

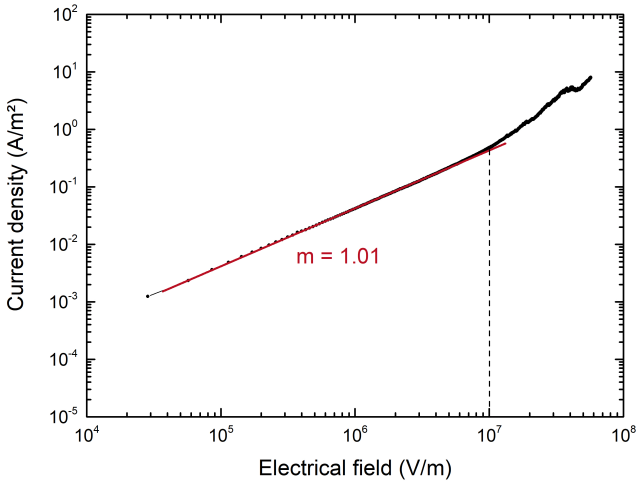

(a) Negative polarity, Ohm fitting

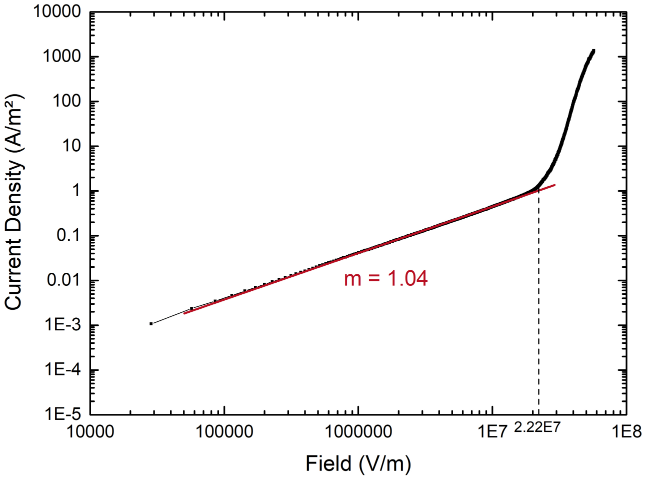

(b) Positive polarity, Ohm fitting

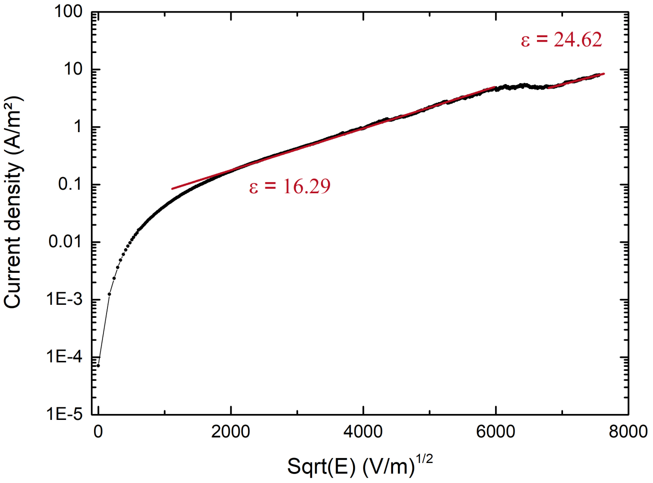

(c) Negative polarity, Schottky fitting

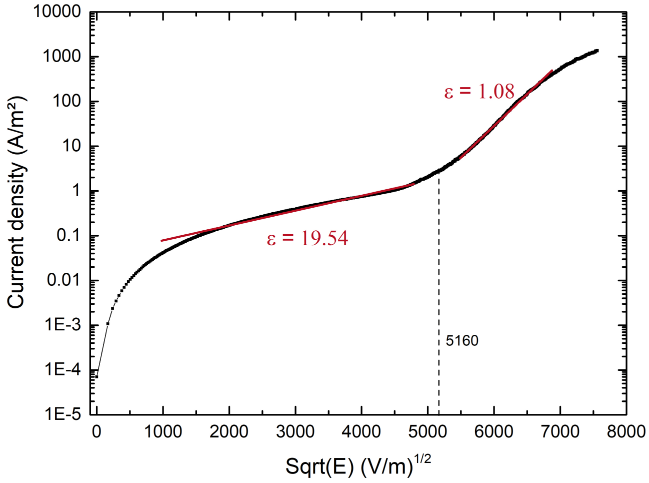

(d) Positive polarity, Schottky fitting

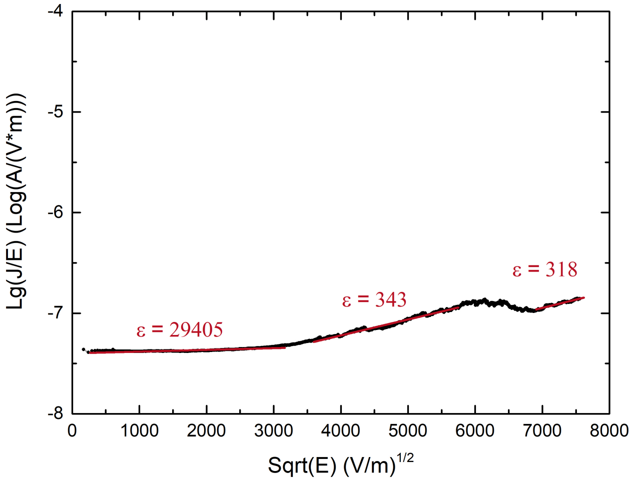

(e) Negative polarity, Poole-Frenkel fitting

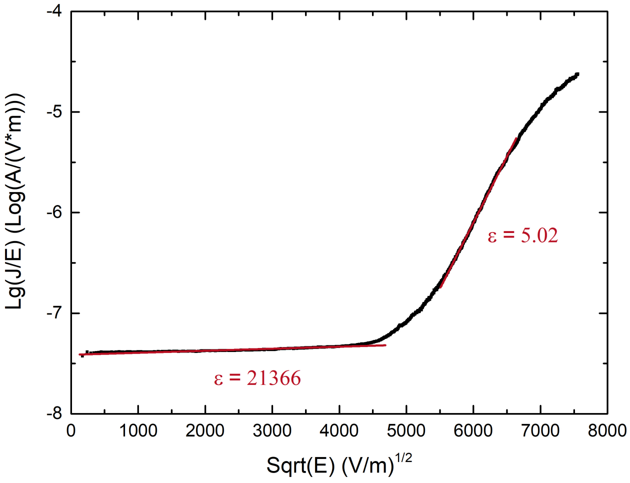

(f) Positive polarity, Poole-Frenkel fitting

Figure S8: Leakage current fittings for the $NaNbO_3$ thin film annealed with $p(O_2) = 100\,\text{mbar}$.

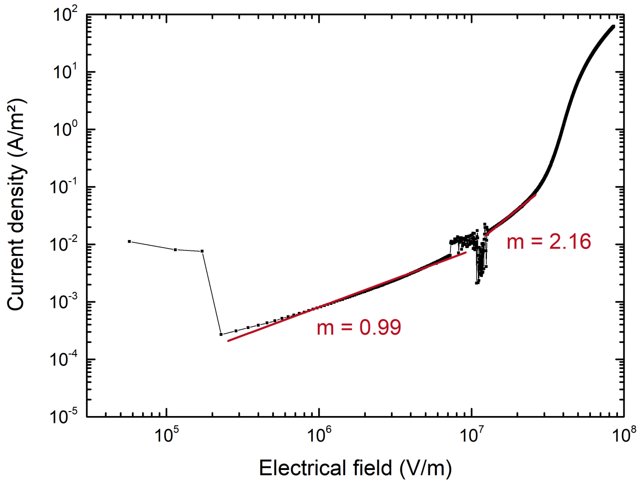

(a) Negative polarity, Ohm fitting

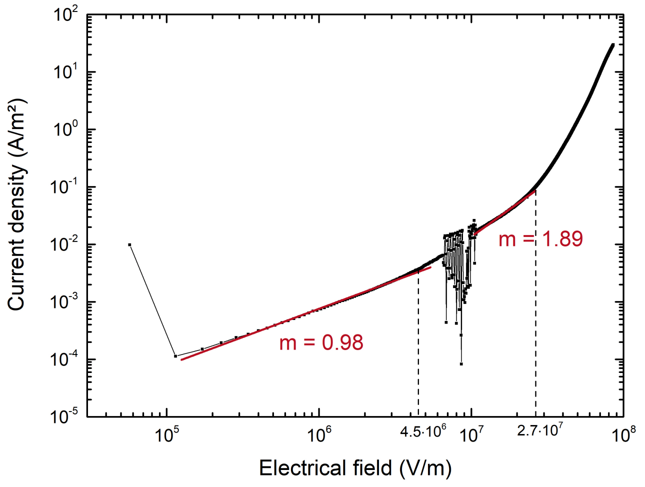

(b) Positive polarity, Ohm fitting

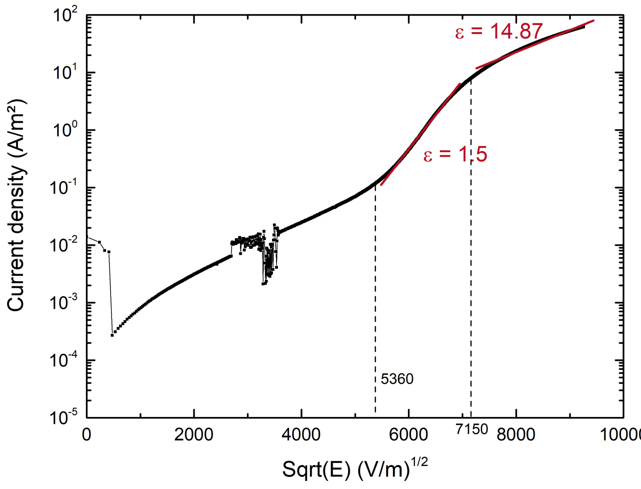

(c) Negative polarity, Schottky fitting

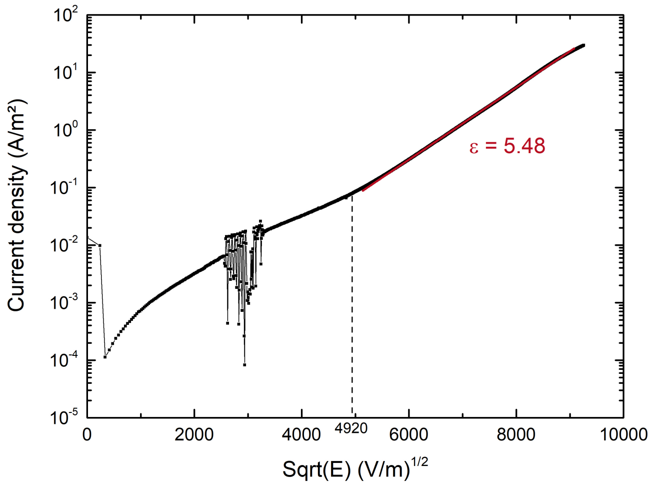

(d) Positive polarity, Schottky fitting

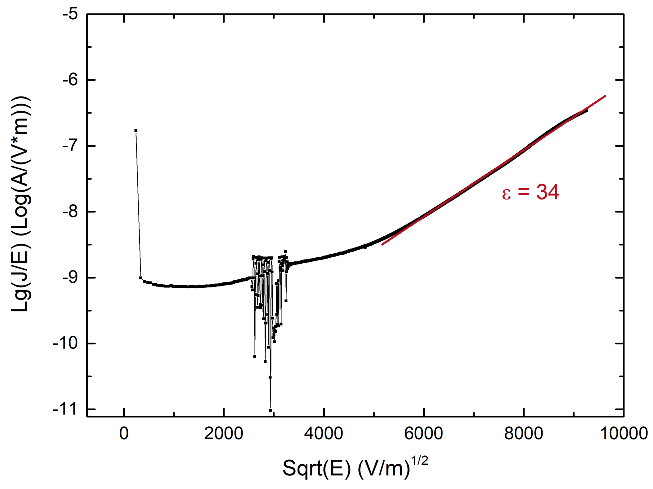

(e) Negative polarity, Poole-Frenkel fitting

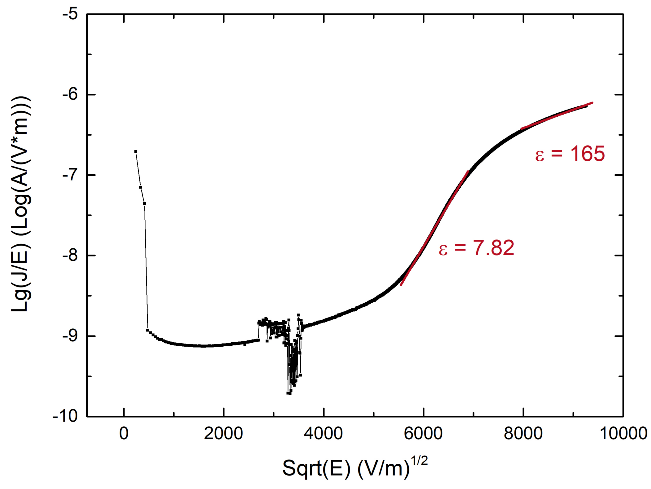

(f) Positive polarity, Poole-Frenkel fitting

Figure S9: Leakage current fittings for the $NaNbO_3$ thin film annealed with $p(O_2) = 350\,\mathrm{mbar}$.

Evaluation of the impact of the heat treatment on structural properties

The impact of the heat treatment on the structural properties like crystal structure and strain has been investigated to ensure that no relaxation or phase changes occur alongside the reduction of the leakage current. For all films which were investigated in this regard, no significant changes were visible neither to the crystal structure nor to the strain state. Exemplary the before versus after XRD $\theta - 2\theta$ scans of the thinnest and thickest sample on $SrTiO_3$ are shown in Figure S10. Only minor changes are recognizable in the Laue oscillations for the thinnest sample, and the peak position of the $NaNbO_3$ 004 reflex for the thickest sample. The latter moves to higher angles after the annealing, which could be caused by the decrease of defects due to the heat treatment. For the comparison of the RSMs, the 310 nm thick $NaNbO_3$ film on $SrTiO_3$ is shown in Figure S11. No impact on the strain state or the superlattice peaks is recognizable, only a broadening of the substrate reflex in Q_x direction, which should not affect the film properties.

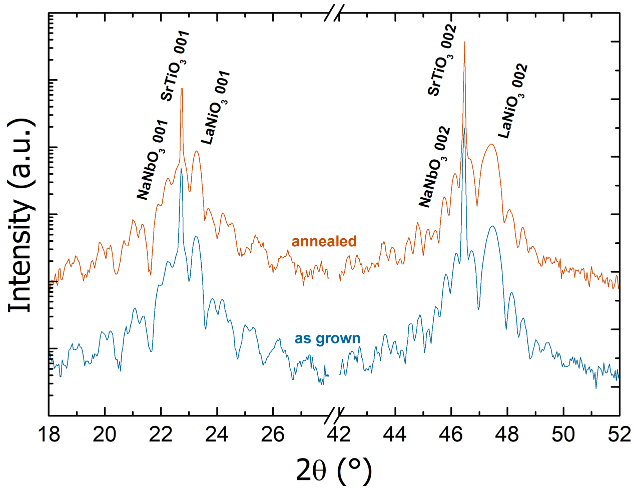

(a) 10 nm thick $NaNbO_3$ film on $SrTiO_3$.

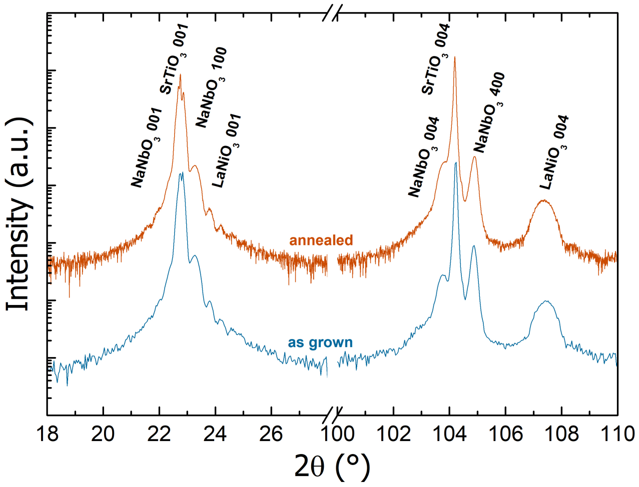

(b) 310 nm thick $NaNbO_3$ film on $SrTiO_3$.

Figure S10: $\theta - 2\theta$ scans on the same sample before and after the heat treatment.

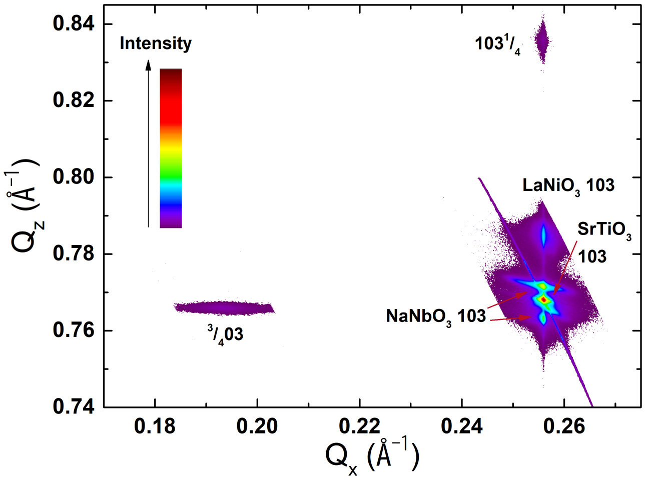

(a) RSM before the heat treatment.

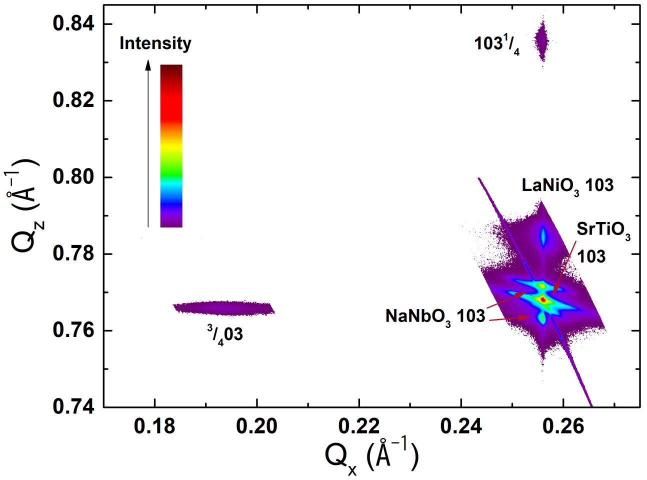

(b) RSM after the heat treatment.

Figure S11: RSM of the 310 nm thick $NaNbO_3$ film on $SrTiO_3$ before and after the heat treatment.

References

(1) Schneider, T.; Cardoletti, J.; Ding, H.; Zhang, M.-H.; Jiang, T.; Major, M.; Komissinskiy, P.; Molina-Luna, L.; Alff, L. Evidence for antipolar displacements in $NaNbO_3$ thin films. *Appl. Phys. Lett.* **2022**, *121*, 122906.

(2) Chiu, F.-C. A Review on Conduction Mechanisms in Dielectric Films. *Adv. Mater. Sci. Eng.* **2014**, *2014*, 1–18.

(3) Zhou, J.; Sando, D.; Cheng, X.; Ma, Z.; Valanoor, N.; Zhang, Q. Tuning Phase Fractions and Leakage Properties of Chemical Solution Deposition-Derived Mixed-Phase $BiFeO_3$ Thin Films. *ACS Appl. Electron. Mater.* **2020**, *2*, 4099–4110.

(4) Rose, A. Space-Charge-Limited Currents in Solids. *Phys. Rev.* **1955**, *97*, 1538–1544.

(5) Singh, J. K.; Mandal, S. K.; Banerjee, G. Refractive index of different perovskite materials. *J. Mater. Res.* **2021**, *36*, 1773–1793.
